# Time-dependent haemoperfusion after acute paraquat poisoning

**DOI:** 10.1038/s41598-017-02527-0

**Published:** 2017-05-22

**Authors:** Hao-Ru Wang, Jian Pan, An-Dong Shang, Yuan-Qiang Lu

**Affiliations:** Department of Emergency Medicine, First Affiliated Hospital, School of Medicine, Zhejiang University, Hangzhou, 310003 People’s Republic of China

## Abstract

Early haemoperfusion (HP) therapy has been found to be very effective in acute paraquat (PQ) poisoning, but the effective rescue window is still uncertain. Demographic data and the type of therapies administered of 621 patients were included as confounding factors in this retrospective study. After receiver operating characteristic curve analysis and intra-group/subgroup analysis, the initiation of glucocorticoid therapy within 3 hrs of exposure with a second treatment given <21 hrs after exposure, HP initiated within 4 hrs of exposure with a second treatment given <20 hrs after exposure, the appearance of pulmonary lesions ≤8 days after exposure and six other variables were used in a multiple analysis. The strength of positivity of the PQ urine test on admission, HP initiated within 4 hrs of exposure with a second treatment given <20 hrs after exposure, the appearance of pulmonary lesions ≤8 days after exposure, and stage III AKI on admission were independent factors of survival probability. HP therapy for acute PQ poisoning was the main therapeutic intervention investigated in this study. Outcomes were time dependent, and the crucial factor was the initiation of therapy within 4 hrs of PQ poisoning followed by a second treatment within 20 hrs.

## Introduction

Paraquat (N,N′-dimethyl-4,4′-bipyridinium dichloride, PQ) is a widely used herbicide in some Asian countries due to its high efficiency and relatively low cost. However, accidental PQ poisoning is a serious health problem associated with a mortality rate of 60–70%^1–5^. Reliable prognostic factors would be helpful in guiding treatment, and early prediction of inevitable death would be important in avoiding inappropriate treatment in patients with acute PQ poisoning^6^.

Survival in cases of PQ poisoning have been described as dose and time dependent^7^. In particular, the PQ concentration in body fluid will reach its maximum level in the first 4–5 hrs^8^. However, reliance on mortality predictions of almost 20% is unlikely to be the optimal method^5^. PQ intoxication thus frequently causes death due to respiratory and kidney failure^9, 10^. Early initiation of treatment is the most important factor in survival, and renal protection is the cornerstone of treatment^11^. The reduction rate of PQ concentration by haemoperfusion (HP) is 67–83% in three hours^12^, so HP is very effective in acute poisoning rescue^13^.

As a provincial centre for PQ intoxication treatment, our facility has been treating approximately 300 cases annually. Patients are sent to our centre directly or transferred from other hospitals, with or without first aid after PQ poisoning. We assumed that the effect of HP was dependent on the rescue window and that the window was likely influenced by many factors. In this article, we performed a retrospective study to review patient survival conditions six months after PQ poisoning and to investigate our assumptions about the therapeutic window for HP therapy.

## Results

There were 705 patients initially reviewed. After further screening, 84 patients were excluded: 24 were younger than 18 years of age. 23 generated negative results from serial PQ semiquantitative urine testing conducted three times on three different days, 8 were discharged within 24 hrs without any further treatment, 9 could not estimate the exact time of poisoning, and 20 were ‘lost’ and could not be followed up.

Finally, a total of 621 patients were used for analysis; 327 (52.66%) of these survived 6 months after PQ poisoning. The mean age of the full group was 37.05 ± 13.27 years. Of the patients, 298 were males, and 323 were females. One hundred seventy-three (27.86%) patients were directly taken to our institute (group A), 426 (68.60%) were transferred to us after first aid (group B), and 22 (3.54%) were transferred from more than one hospital (group C). One hundred forty-two (32.47%) patients had a medical history, and 63 (14.41%) took long-term medication. Demographic data and univariate analyses comparing patient survival and patient death are shown in Table 1.

**Table 1:** Demography data and univariate analysis between survival and death patients.

Variables	Survival Patients (numbers/mean ± SD)		*t*/*χ* ^2^ value	*P* value
	Yes	No		
Age (years)	34.82 ± 10.23	39.28 ± 15.79	1.024	0.121
Gender(Male/Female)	159/168	139/155	2.156	0.113
Arrival type				
Group A	227	199	1.852	0.043
Group B	81	92		
Group C	8	14		
Medical history (Y/N)	142/185	137/157	2.417	0.108
Hypertension	53	69	2.325	0.112
Asthma	8	21	2.964	0.048
COPD	12	35	3.112	0.043
Bronchiectasis	9	17	2.318	0.120
Coronary heart disease	38	79	1.321	0.316
DM	11	26	0.954	0.793
Congenital heart disease	6	12	1.021	0.112
Tumor	5	7	0.527	0.931
Medication history	63/264	78/216	2.376	0.137
Glucocorticoid	12	26	1.853	0.115
Immunosuppressive drugs	3	8	3.685	0.026
Bronchodilators	14	22	1.225	0.351
Anticoagulants	34	49	1.014	0.462
Semiquantitative of PQ level at admission				
+	168	124	2.736	0.036
++	133	118		
+++	26	52		
Gastric lavage (Y/N)	249/78	207/87	1.051	0.423
Cathartic (Y/N)	169/158	145/149	0.825	0.781
Emetic (Y/N)	49/278	51/243	1.437	0.165
Pulmonary lesions (Y/N)	127/200	118/176	2.225	0.041
Serum creatinine at admission (mg/dL)	0.76 ± 0.19	1.01 ± 0.17	3.701	0.045

Group A: Patients were directly taken to our institute. Group B: Patients were transferred to us after first aid. Group C: Patients were transferred from more than one hospital. COPD: chronic obstructive pulmonary disease. DM: diabetes mellitus.

There were significant differences in the survival rate based on the number of positive PQ semiquantitative urine tests (positive result at admission: *χ* ^2^ = 2.736, *P* = 0.036; positive results at admission and first repeat test: *χ* ^2^ = 3.108, *P* = 0.031; all three positive results: *χ* ^2^ = 3.452, *P* = 0.022), the arrival type (survival rate of group A, B and C were 53.07%, 51.96%, and 48.65%, respectively, *χ* ^2^ = 1.852, *P* = 0.043), a medical history of chronic obstructive pulmonary disease (COPD) or asthma (*χ* ^2^ = 3.112, *P* = 0.043; *χ* ^2^ = 2.964, *P* = 0.048), a medication history positive for immunosuppressive drugs (*χ* ^2^ = 3.685, *P* = 0.026), serum creatinine on admission (*t* = 3.701, *P* = 0.045) and acute kidney injury (AKI) stage I, II or III (*χ* ^2^ = 13.152, *P* = 0.008). Glucocorticoids, immunosuppressors and HP were used a median of 7, 8 and 10 times, respectively. No significant difference was found between the survival rate and the number of repeated treatments (*χ* ^2^ = 0.871, *P* = 0.135). Serum creatinine tests were conducted 8 times/10 days (range 7–16 days). It was first tested on admission, and testing was repeated after each HP treatment. There was a significant difference between serum creatinine levels on admission and after HP (*F* = 23.207, *P* = 0.034), but no significant difference was found after glucocorticoid or immunosuppression therapy. In addition, no significant difference in survival rate was found between age, gender, and other demographic data.

The time after PQ poisoning was considered in further analysis. Patients reached our institute at a mean time of 5.84 ± 2.37 hrs. The mean time of groups A, B and C were found to have a significant difference (2.95 ± 1.96 hrs, 6.97 ± 2.62 hrs and 8.12 ± 3.63 hrs respectively, *t* = 4.371, *P* = 0.024). The mean time to semiquantitative urine test after initial PQ exposure was 5.84 ± 2.37 hrs for the first test, 10.97 ± 3.45 hrs for the second test and 17.24 ± 4.06 hrs for the third test. Gastric lavage, cathartics, and emetics were used only once; the mean time to each treatment was 2.46 ± 2.18 hrs, 3.28 ± 3.02 hrs and 1.54 ± 1.17 hrs, respectively. A significant difference was found in the first appearance of AKI stage I (*P* = 0.036), the first appearance of pulmonary lesions (*P* = 0.024), the first use of glucocorticoids (*P* = 0.043), and the first and second uses of HP (*P* = 0.033) (Table 2). No significant difference was found between patient survival and patient death for the mean time to gastric lavage (*P* = 0.106), cathartic administration (*P* = 0.173), or emetic administration (*P* = 0.152). Additionally, no significant correlation was found between survival rate and the time to gastric lavage (Pearson’s correlation = 1.203, *P* = 0.091), cathartic administration (Pearson’s correlation = 0.357, *P* = 0.114) or emetic administration (Pearson’s correlation = 0.211, *P* = 0.285).

**Table 2:** Time difference between survival and death patients within tests and therapies after PQ poisoning.

Variables	Survival Patients (mean ± SD, hrs)		*t*/*χ* ^2^ value	*P* value
	Yes	No		
The first appearance of AKI				
Stage I	7.25 ± 1.96	3.78 ± 1.24	4.326	0.036
Stage II	35.39 ± 8.14	27.65 ± 9.33	3.081	0.058
Stage III	39.61 ± 12.42	34.30 ± 13.28	2.764	0.092
The first appearance of pulmonary lesions (Y/N)	203.13 ± 50.61	168.57 ± 46.75	4.029	0.024
Gastric lavage (Y/N)	2.21 ± 1.07	3.37 ± 2.49	2.062	0.106
Cathartic (Y/N)	3.03 ± 2.94	5.16 ± 3.15	1.331	0.173
Emetic (Y/N)	1.28 ± 1.09	3.26 ± 1.53	1.824	0.152
Glucocorticoid				
TG1	3.01 ± 2.05	3.52 ± 2.49	2.797	0.005
TG2	27.63 ± 6.35	29.01 ± 7.32	2.515	0.012
TG3	50.26 ± 7.17	51.73 ± 8.29	2.370	0.018
TG4	73.35 ± 7.35	74.22 ± 8.48	1.374	0.170
TG5	96.85 ± 8.79	97.93 ± 9.25	1.491	0.136
TG6	120.72 ± 9.93	121.69 ± 9.26	1.255	0.210
TG7	143.85 ± 10.78	144.36 ± 11.19	0.581	0.563
Immunosuppressor				
TI1	3.55 ± 2.24	3.93 ± 2.68	−1.923	0.055
TI2	27.41 ± 7.36	28.76 ± 10.57	−1.769	0.063
TI3	52.01 ± 7.84	51.87 ± 10.96	0.601	0.548
TI4	75.63 ± 8.07	75.15 ± 11.27	1.886	0.060
TI5	98.74 ± 8.12	98.93 ± 11.35	0.732	0.465
TI6	122.26 ± 8.52	122.35 ± 11.48	0.320	0.749
TI7	145.39 ± 9.01	145.52 ± 11.94	0.407	0.684
TI8	168.18 ± 9.37	168.45 ± 12.27	0.777	0.437
Haemoperfusion				
TH1	3.85 ± 3.09	4.53 ± 4.10	2.348	0.020
TH2	23.74 ± 13.21	27.02 ± 14.52	2.948	0.003
TH3	46.63 ± 13.58	46.63 ± 13.58	2.269	0.024
TH4	69.87 ± 14.13	72.08 ± 15.21	1.877	0.061
TH5	93.15 ± 14.92	95.28 ± 16.03	1.715	0.087
TH6	116.48 ± 15.51	118.75 ± 16.89	1.746	0.081
TH7	139.07 ± 16.14	141.69 ± 17.53	1.939	0.053
TH8	162.37 ± 17.08	163.59 ± 18.04	0.865	0.387
TH9	185.74 ± 17.76	186.32 ± 18.69	0.396	0.692
TH10	208.06 ± 18.34	209.81 ± 19.10	1.164	0.245

AKI: Acute kidney injury. TG1, TG2…TG7: the first, second…seven time use of glucocorticoid. TI1, TI2…TI8: the first, second…eight time use of immunosuppressor. TH1, TH2…TH10: the first, second…ten time use of haemoperfusion.

**Figure 1:**
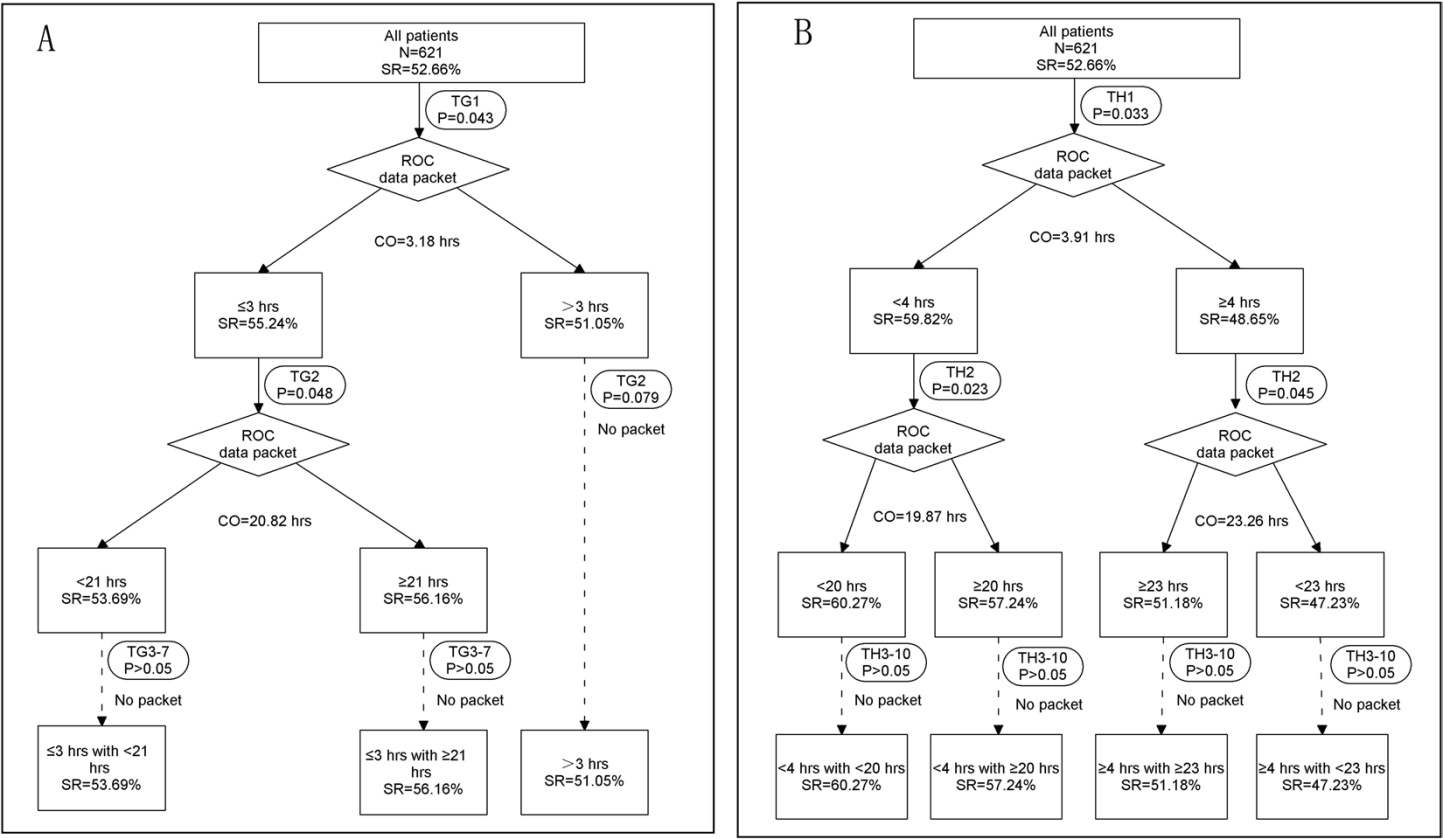
Steps of data packet after ROC curve analysis. TG: Time of glucocorticoid; TH: Time of haemoperfusion; CO: Cut-off time; SR: Survival rate; ROC: Receiver operating characteristic.

Glucocorticoid therapy, immunosuppression therapy and HP were provided approximately once daily; glucocorticoids were the only therapy given before transfer to our institute. No significant difference was found between survival rate and repeated therapy administrations (number of glucocorticoid treatments: *χ* ^2^ = 0.871, *P* = 0.135, number of immunosuppressor treatments: *χ* ^2^ = 0.914, *P* = 0.248, times of HP: *χ* ^2^ = 1.014, *P* = 0.102). Therefore, the variables were expressed in terms of hours (hours of glucocorticoid therapy: TG1 to TG7, hours of immunosuppressor therapy: TI1 to TI8, hours of HP: TH1 to TH10) and used as dummy variables (Table 2). For those with a first appearance of stage I AKI, the number of pulmonary lesions present was significantly different in cases of survival and cases of death. A significant difference was also found in patient survival and patient death between the first, second and third administration times of both glucocorticoids and HP. After receiver operating characteristic (ROC) curve analysis, the cut-off time between patient survival and patient death from the first appearance of pulmonary lesions was 196.83 hrs (rounded to 8 days). Variable pulmonary lesions were categorized by the time of the first appearance, ≤8 days, >8 days or no appearance. The cut-off times to first administration of glucocorticoid, immunosuppressor and HP were 3.18 hrs (rounded to 3 hrs), 3.91 hrs (rounded to 4 hrs), respectively, as shown in Fig. 1. The glucocorticoid variable of was divided into two groups, ≤3 hrs and >3 hrs (survival rates were 55.24% and 51.05%, respectively). The HP variable was also divided into two groups, <4 hrs and ≥4 hrs (survival rates were 59.82% and 48.65%, respectively). A significant difference after the second glucocorticoid treatment was found in the ≤3 hrs subgroup (mean time of patient survival 28.29 ± 6.73 hrs versus 32.17 ± 8.25 hrs, *t* = 2.814, *P* = 0.048) but not in the >3 hrs subgroup (31.16 ± 8.39 hrs versus 35.04 ± 9.67 hrs, *t* = 2.016, *P* = 0.079). In addition, a significant difference after the second HP treatment was found in the <4 hrs subgroup (31.52 ± 7.90 hrs versus 36.12 ± 6.71 hrs, *t* = 3.791, *P* = 0.023) and in the ≥4 hrs subgroup (37.52 ± 8.53 hrs versus 46.12 ± 9.26 hrs, *t* = 2.868, *P* = 0.045). No significant difference was found in the time of the second immunosuppressor treatment (28.32 ± 2.34 hrs versus 33.19 ± 4.81 hrs, *t* = 5.261, *P* = 0.39). After ROC curve analysis, the cut-off for the second glucocorticoid treatment was 20.82 hrs (rounded to 21 hrs) in the ≤3 hrs subgroup, and the cut-off for the second HP treatment was 19.87 hrs (rounded to 20 hrs) in the <4 hrs subgroup and 23.26 hrs (rounded to 23 hrs) in the ≥4 hrs group. The glucocorticoid variable of was divided into three groups: (i) first treatment ≤3 hrs, second treatment <21 hrs, (ii) first treatment ≤3 hrs, second treatment ≥21 hrs and (iii) first treatment >3 hrs (survival rates were 56.16%, 53.69% and 51.05%, respectively). The HP variable was divided into four groups: (i) first treatment <4 hrs with second treatment <20 hrs, (ii) first treatment <4 hrs with second treatment ≥20 hrs, (iii) first treatment ≥4 hrs with second treatment <23 hrs and (iv) first treatment ≥4 hrs with second treatment ≥23 hrs (survival rates were 60.27%, 57.24%, 51.18 and 47.23%, respectively). No significant difference between patient survival and patient death was found in either of these subgroups or in other time periods of glucocorticoid, immunosuppressor or HP treatment (all *P* > 0.05).

After multiple logistic regression analysis, we found the independent risk factors were semiquantitative urine PQ test level of ++ on admission (*P* = 0.023), semiquantitative urine PQ test level of +++ (*P* = 0.001), first HP <4 hrs with second HP <20 hrs (*P* = 0.003), and first appearance of pulmonary lesions ≤8 days (*P* = 0.047) (Table 3). The ratio of survival rates between the first and second HP treatments was 1.6 versus 1.

**Table 3:** Multiple logistic regression analysis to identify independent factors of HP therapy survival probability.

Variables	B	Std. Coefficients	Std. Error	*t* value	*P* value
Semiquantitative of PQ level at admission					
++	−0.067	−0.141	0.029	−2.292	0.023
+++	−0.084	−0.211	0.023	−3.645	0.001
Arrival type					
Group B	−0.023	−0.053	0.021	1.306	0.193
Group C	−0.024	−0.075	0.030	0.930	0.353
Medication history of asthma (Y/N)					
Asthma	−0.029	−0.094	0.019	−1.669	0.096
COPD	−0.059	−0.102	0.017	−1.857	0.082
Medication history of Immunosuppressive drugs (Y/N)	0.055	0.085	0.037	1.495	0.136
Pulmonary lesions					
The first appearance >8 days	−0.232	−0.318	0.104	1.015	0.055
The first appearance ≤8 days	−0.133	−0.274	0.085	1.457	0.047
Haemoperfusion first time <4 hrs with second time <20 hrs	−0.138	−0.357	0.023	−7.354	0.004
Glucocorticoid used first time ≤3 hrs with second time <21 hrs	−0.113	−0.125	0.022	−1.495	0.127
AKI at admission					
Stage II	−0.048	−0.105	0.022	1.608	0.054
Stage III	−0.072	−0.172	0.054	2.213	0.028

AKI: Acute kidney injury. HP: haemoperfusion. Group B: Patients were transferred to us after first aid. Group C: Patients were transferred from more than one hospital.

## Discussion

This study found that lower urine dithionite PQ and a time to first HP treatment of <4 hrs with a time to second treatment of <20 hrs were independent predictors for PQ poisoning survival. Earlier studies identified significant prognostic factors such as age, the amount of PQ ingested, plasma PQ concentration, and renal function^3, 14^. Reliable predictors of prognosis would be helpful in guiding therapy. Previous studies considered plasma PQ concentration to be a marker of severity and prognosis^7, 15–17^. However, none of these as yet alter clinical management; they are all time-dependant prediction methods^5^. In this study, we were able to elucidate the potential benefit of providing treatment in a finite period instead of relying on predictions.

Severe secondary AKI occurred in approximately 50% of cases of PQ intoxication^18^. The key to managing AKI is ensuring adequate renal perfusion by achieving and maintaining haemodynamic stability while avoiding hypovolemia^19^. Studies had suggested that performing the initial HP before the PQ level reached its peak would be the most effective way of eliminating PQ from the body^12^, noting that the plasma PQ level usually peaks within one hour of PQ ingestion^11^. A research study revealed that early HP after PQ exposure might be effective in reducing mortality^20^. However, we were not sure if HP performed within one hour of ingestion would be more helpful since there were almost no patients who arrived within that time period. Despite the fact that the urine dithionite PQ test has low sensitivity, it is still a useful bedside screening tool for PQ intoxication because of its convenience and reproducibility^11^. It was also found that the time to achieve a negative urine dithionite test is a reliable marker for predicting mortality^21^. We could not identify the exact amount of time required for the semiquantitative urine test to turn negative since there was no further testing after treatment. We presumed that patients would achieve a negative test sooner if HP was performed for first time <4 hrs after ingestion with the second treatment given <40 hrs post ingestion. The second HP might be helpful in eliminating the catabolite produced by oxygen free radical damage.

The key to rescue therapy is the time to administration of appropriate therapy when there is no specific antidote available. Medical management with gastrointestinal decontamination techniques, methods to increase poison elimination, proper hydration and supportive management are the most important factors in the survival rate in cases of PQ poisoning^22^. The sooner gastrointestinal decontamination is performed, the better is the outcome^23^. A beneficial effect was found when gastric lavage was performed <4 hrs post ingestion, but it appeared to adversely affect the outcome if performed more than 4 hrs post exposure^5^. Activated charcoal seemed useful in the early stages of acute self-poisoning, but no substantiating evidence has been found^24^. The use of activated charcoal in PQ poisoning has not been reported in the literature; further study to assess the effectiveness of activated charcoal in PQ poisoning is still needed. Adverse effects of gastric lavage have been reported—it may have led to aspiration, asphyxia or mediastinal perforation^25, 26^. However, no direct complication attributable to gastric lavage was found in our study.

On-line high-volume HP can rapidly clear inflammatory cytokines, reduce systemic inflammatory response syndrome, and improve the survival of patients poisoned with organophosphorus pesticides^13^. Similarly, continuous venovenous haemodiafiltration was successfully used in the rescue of a patient with a high dose PQ exposure of 36.48 mg/kg^21^. Thus, more patients might be saved if PQ elimination or renal replacement therapy can be more efficiently administered a short time after poisoning.

There were several limitations to this study. First, it was not possible to obtain measurements of the change in PQ concentration after HP. In addition, the effect of this change on the survival rate is unknown. Second, HP may be influenced by the level of oxidative stress in patients, and this was not estimated in this study. Third, although various renal replacement techniques have been shown to improve outcomes for patients with severe poisoning, further study is required to ascertain the specific effects of these techniques in cases of PQ poisoning.

Overall, the effectiveness of HP therapy in PQ poisoning was time dependent and helpful in the rescue window defined as a time to first use within 4 hrs of exposure and a time to second use within 20 hrs. Moreover, the crucial factor was processing HP within 4 hrs of PQ intoxication. The sooner the treatment the better. Further study is needed to ensure the rescue window will last longer if first aid is performed correctly and in a timely manner.

## Methods

**Figure 2:**
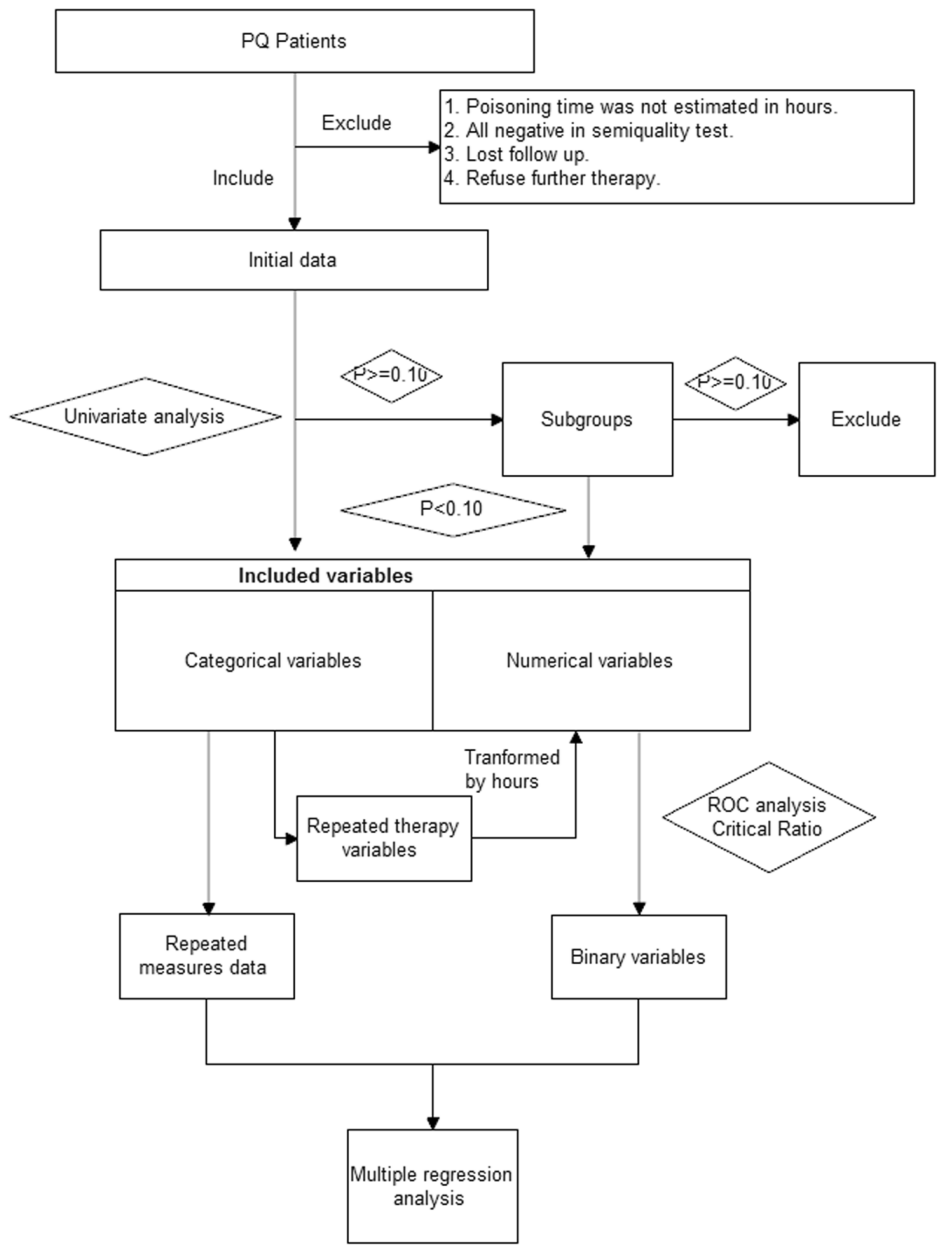
Flowchart of the study protocol.

We collected data on patients with PQ poisoning treated at our institute between May 2002 and April 2012. The inclusion criteria included adulthood (18 years and over) and positive PQ urine semiquantitative test. The exclusion criteria were age less than 18 years of age, lost to follow-up, suspected cases ruled out (all three PQ urine semiquantitative tests were negative), unknown time of exposure, and discharge within 24 hrs without any further treatment. The patient flowchart and the study protocol are shown in Fig. 2.

All dependent variables and subgroups were determined by univariate analysis. Subgroups contained the therapies provided each hour and the number times each therapy was repeated. The data analysis included arrival time (directly arrived or transferred), age, gender, medical history [diabetes mellitus, hypertension, coronary heart disease, congenital heart disease, COPD, asthma and tumour], medication history longer than 6 months (glucocorticoid, immunosuppressive drugs, bronchodilators, anticoagulants), semiquantitative urine dithionite PQ test results (the first test was performed on admission; subsequent tests were given at 6-hr intervals, normally 3–4 times), gastrointestinal decontamination (gastric lavage, cathartic administration, and emetic administration), use of glucocorticoids and immunosuppressive drugs, HP (average reinfusion rate was 9 L/session and 300 mL/min in the postdilution mode), serum creatinine (the first blood test was performed on admission, tested daily for the first 3 days and then tested at 1- to 2-day intervals), and pulmonary lesions. Changes in serum creatinine level categorized AKI into three stages (increase ≤0.3 mg/dL, 0.3–4 mg/dL and ≥4 mg/dL)^19^. Pulmonary lesions were diagnosed by characteristic effusion and fibrosis in computed tomography (CT) images (the first CT scan was performed on admission, subsequent CT scans were performed at 2- to 3-day intervals). The number of times each therapy was repeated, the frequency of outcome measurements, and the rescue window after PQ poisoning were assessed in hours.

Student’s *t* test or a Wilcoxon rank-sum test was performed on numerical data among groups. The chi-square test was used for categorical data. Pearson correlation analysis or curve estimation was used to estimate correlations between two variables. Repeated-measures analysis of variance was used to estimate the relationship between repeated therapies and the tests. ROC curve analysis was used to evaluate the threshold value of numerical variables, which were then transformed into binary variables. A one-way analysis of variance or Kruskal-Wallis test was used to compare multiple subgroups; otherwise, subgroups were divided into dummy variables and analysed by univariate analysis. The variables with *P* values less than or equal to 0.10 were used in the multiple-factor analysis. Multiple logistic regression analysis was used to evaluate the relationship between dependent variables and survival probability. The level of statistical significance was set at *P* < 0.05. All analyses were performed using SPSS software (version 13.0, SPSS Inc., IBM, Chicago, IL, USA).

This study was approved by the Ethical Committee of the First Affiliated Hospital, School of Medicine, Zhejiang University. We obtained the verbal consent of all patients or their relatives by phone calls.
